# Complete chloroplast genome of *Angelica keiskei* (Umbelliferae)

**DOI:** 10.1080/23802359.2021.1873711

**Published:** 2021-02-11

**Authors:** Wen-Jie Yan, Tian-Ge Yang, Rui Qin, Hong Liu

**Affiliations:** a College of Biochemical Engineering, Beijing Union University, Beijing, China; b Hubei Provincial Key Laboratory for Protection and Application of Special Plant Germplasm in Wuling Area of China, Key Laboratory of State Ethnic Affairs Commission for Biological Technology, College of Life Science, South-Central University for Nationalities, Wuhan, China

**Keywords:** *Angelica keiskei*, Apiaceae, chloroplast genome

## Abstract

*Angelica keiskei* (Miq.) Koidz. is a perennial herbaceous plant belonging to the genus *Angelica*, Umbelliferae. As a plant with dual-purpose as food and medicine, it has the potential for the future development of high-value functional products. The complete chloroplast genome has a total size of 147,007 bp, consisting of two inverted repeats (IR, 18,508 bp, each), and separated by a large single-copy region (LSC, 92,415 bp) and a small single-copy region (SSC, 17,576 bp). Further annotation revealed the chloroplast genome contains 128 genes, including 84 protein-coding genes (80 PCG species), 36 tRNA genes (30 tRNA species), and 8 rRNA genes (4 rRNA species). A total of 83 simple sequence repeats (SSRs) were identified in the chloroplast genome. This chloroplast genome resource will be useful for the study of the evolution and genetic diversity of *Angelica keiskei* in the future.

*Angelica keiskei* (Miq.) Koidz. is a large perennial herb plant belonging to the genus *Angelica*, Umbelliferae, and mainly distributed in Central and Western China. Most plants in this genus are frequently used as folk medicine. The roots of *A. keiskei* are used in traditional Chinese medicine for replenishing blood, treating abnormal menstruation, and other diseases associated with women’s reproductive health (Chen et al. 2013). *Angelica keiskei* could help to prevent thrombotic diseases and has become popular as herbal medicine, dietary supplement, and health food in Asian countries (Ohkura et al. 2018). In this study, to obtain new insights into the phylogeny of *A. keiskei*, we sequenced, assembled, and annotated the accurate chloroplast genome.

The *A. keiskei* whole herb specimen was collected from Huangpi Medicinal Herb Garden, Wuhan, Hubei province of China (WH2019120508946, 114°18′58′′E, 30°34′51′′N). The voucher specimens were deposited at the Herbarium of South-Central University for Nationalities (HSN). The complete genomic DNA was extracted using CTAB method (Doyle and Doyle 1987) and sequenced using the Illumina NovaSeq platform at Majorbio Company (Shanghai, China). A total of 2.8 Gb raw reads were generated and low-quality sequences were filtered out. The trimmed reads were assembled using GetOrganelle (Jin et al. 2020). The assembled genome was annotated using CPGAVAS2 (Shi et al. 2019) and PGA (Qu et al. 2019). The complete chloroplast genome was 147,007 bp (MW125613) and composed of two inverted repeats (IRs) of 18,508 bp each, which divide a large single-copy (LSC) region of 92,415 bp and a small single-copy (SSC) region of 17,576 bp, the average GC content was 37.51%. The chloroplast genomes encoded 128 functional genes, including 84 protein-coding genes (80 PCG species), 36 tRNA genes (30 tRNA species), and 8 rRNA genes (4 rRNA species). A total of 83 SSR markers ranging from mononucleotide to hexa-nucleotide repeat motif were identified in *A. keiskei* chloroplast genome.

In order to explore the phylogenetic relationship of *A. keiskei* within Leguminosae, the complete chloroplast genomes of 32 species from Leguminosae were obtained from the GenBank database, with the *Brassaiopsis hainla* and *Fatsia japonica* as the outgroups, the phylogenetic trees were built from the whole protein-coding gene matrix by maximum-likelihood (ML) and Bayesian inference (BI) (Figure 1). The ML tree was generated using IQ-TREE (Nguyen et al. 2015) based on the best model of TVM + F+R2 and 1000 bootstrap replicates, and BI analysis was performed in MrBayes-3.2.7 (Ronquist et al. 2012). This result showed that the analyzed *A. keiskei* were closer to the species of *A. polymorpha*.

**Figure 1.**
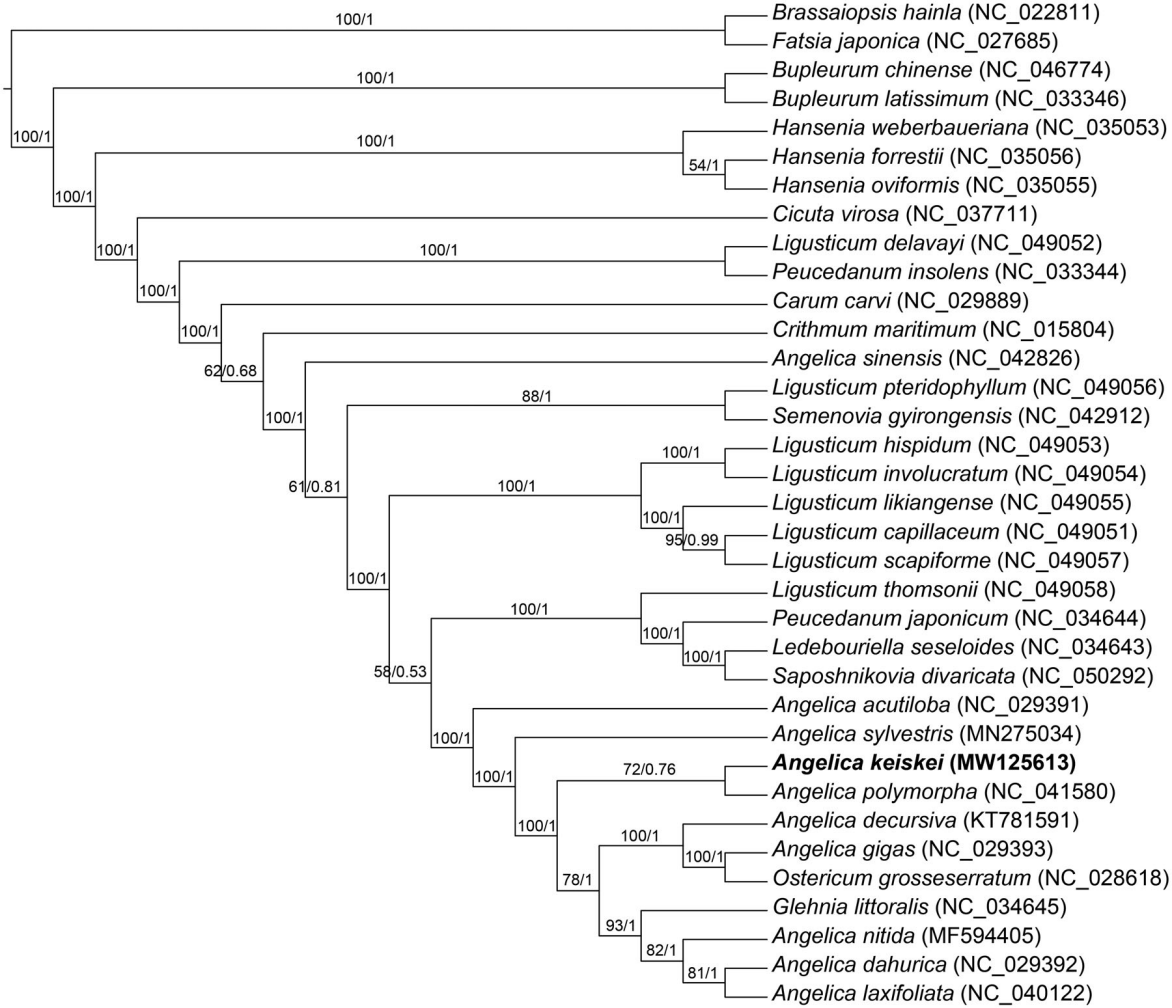
Phylogenetic tree reconstructed by maximum-likelihood (ML) and Bayesian inference (BI) analysis basedon the whole chloroplast protein-coding genes of these 35 species.

This information will be useful for the study of the evolution and genetic diversity of *A. keiskei* in the future.

## Data Availability

The genome sequence data that support the findings of this study are openly available in GenBank of NCBI at [https://www.ncbi.nlm.nih.gov] (https://www.ncbi.nlm.nih.gov/) under the accession no. MW125613. The associated ‘BioProject,’ ‘SRA,’ and ‘Bio-Sample’ numbers are PRJNA670261, SRR12853312, and SAMN16491009 respectively.
